# P-1595. Short Interval Repeat Testing with a Multiplex Pneumonia Panel is Low Yield- A Case for Diagnostic Stewardship

**DOI:** 10.1093/ofid/ofae631.1762

**Published:** 2025-01-29

**Authors:** Eli Wilber, Eric Fitts, Eileen Burd, Lucy S Witt

**Affiliations:** Emory University School of Medicine, Atlanta, Georgia; Emory University, Atlanta, Georgia; Emory University School of Medicine, Atlanta, Georgia; Emory University, Atlanta, Georgia

## Abstract

**Background:**

Multiplex molecular panels have emerged as a tool for the rapid identification of potential pathogens and resistance markers for multiple syndromes including pneumonia. The BioFire® FilmArray® Pneumonia panel (PPP) is a multiplex molecular test that can rapidly detect 33 targets (including bacteria, viruses, and some common resistance genes) from respiratory samples. The role of this test in patient care is evolving and the role of repeat testing is not well described.

**Figure 1:**
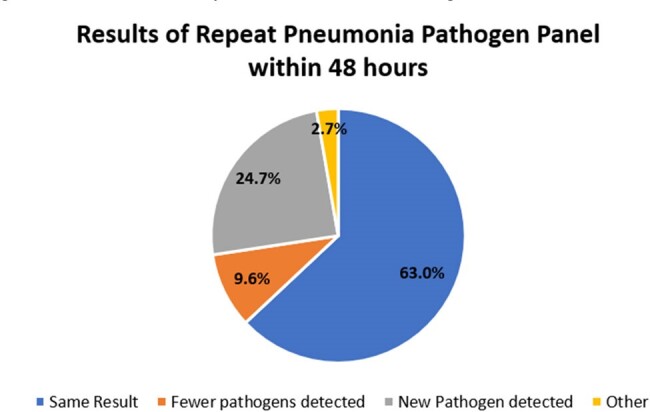
Results of Repeat Pneumonia Pathogen Panel within 48 Hours

**Methods:**

We conducted a retrospective chart review of all PPPs conducted at the Emory University Hospital Clinical Microbiology Lab which supports seven academic-affiliated hospitals during the period 3/13/2023-3/14/2024. Patients with a PPP test result within 48 or 168 hours of a previous test result were identified and reviewed for interval changes in the detected targets.

**Figure 2:**
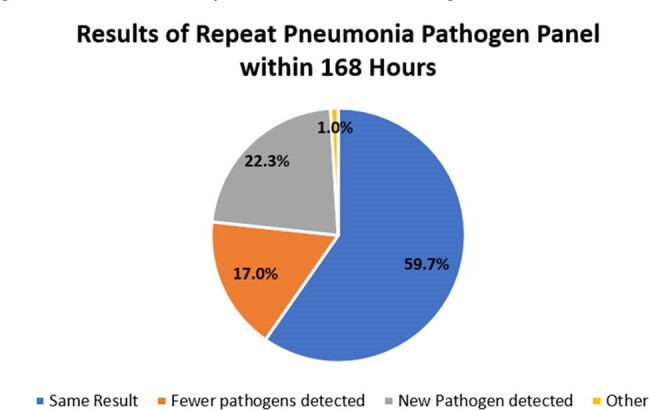
Results of Repeat Pneumonia Pathogen Panel within 168 Hours

**Results:**

There were 2307 PPPs resulted during the study period with a numerical trend of increasing use over time. For the study period, 73 (3.2%) of tests were repeats within 48 hours and 206 (8.9%) were repeats within 168 hours of a previous PPP test. For tests repeated within 48 hours (Figure 1), 46/73 (63.0%) returned the exact same results as the initial test, 7/73 (9.6%) detected fewer pathogens, 18/73 (24.7%) detected at least one additional pathogen. 6/18 (33.3%) of tests detecting a new pathogen detected a common respiratory virus. For tests repeated within 168 hours (Figure 2), 123/206 (59.7%) returned the exact same results, 35/206 (17.0%) detected fewer pathogens, and 45/206 (22.3%) detected at least one additional pathogen.

**Conclusion:**

Repeat testing with the PPP was common during the study period and almost nine percent of the annual testing volume was repeat testing within one week of a previous result. Short interval repeat testing with the PPP typically returns a result with no additional potential pathogens detected and thus may be unlikely to affect clinical management. Ordering restrictions that include a “lockout period” may be a valid diagnostic stewardship approach to improve the use of this test.

**Disclosures:**

**Eli Wilber, MD**, Elsevier: Advisor/Consultant|Roche Diagnostics: Advisor/Consultant

